# Epidemiology of Burns in Rural Bangladesh: An Update

**DOI:** 10.3390/ijerph14040381

**Published:** 2017-04-05

**Authors:** Siran He, Olakunle Alonge, Priyanka Agrawal, Shumona Sharmin, Irteja Islam, Saidur Rahman Mashreky, Shams El Arifeen

**Affiliations:** 1 Department of International Health, Bloomberg School of Public Health, Johns Hopkins University, Baltimore, MD 21205, USA; siranhe@gmail.com (S.H.); pagrawa6@jhu.edu (P.A.); 2 International Center for Diarrheal Disease Research, GPO Box 128, Dhaka 1000, Bangladesh; shumona@icddrb.org (S.S.); irteja.islam@icddrb.org (I.I.); shams@icddrb.org (S.E.A.); 3 Center for Injury Prevention and Research, House # B-162, Road # 23, New DOHS, Mohakhali, Dhaka 1206, Bangladesh; mashreky@ciprb.org

**Keywords:** burns, epidemiology, Bangladesh, risk factors, low and middle-income countries

## Abstract

Each year, approximately 265,000 deaths occur due to burns on a global scale. In Bangladesh, around 173,000 children under 18 sustain a burn injury. Since most epidemiological studies on burn injuries in low and middle-income countries are based on small-scale surveys or hospital records, this study aims to derive burn mortality and morbidity measures and risk factors at a population level in Bangladesh. A household survey was conducted in seven rural sub-districts of Bangladesh in 2013 to assess injury outcomes. Burn injuries were one of the external causes of injury. Epidemiological characteristics and risk factors were described using descriptive as well as univariate and multivariate logistic regression analyses. The overall mortality and morbidity rates were 2 deaths and 528 injuries per 100,000 populations. Females had a higher burn rate. More than 50% of injuries were seen in adults 25 to 64 years of age. Most injuries occurred in the kitchen while preparing food. 88% of all burns occurred due to flame. Children 1 to 4 years of age were four times more likely to sustain burn injuries as compared to infants. Age-targeted interventions, awareness of first aid protocols, and improvement of acute care management would be potential leads to curb death and disability due to burn injuries.

## 1. Introduction

Burns account for approximately 265,000 deaths each year on a global scale, with a huge preponderance in low- and middle-income countries (LMICs) [1]. The death rate due to burns is 11 times higher in LMICs compared to high-income countries (HICs) [2]. Burn injuries mostly occur due to heat transfer from hot liquids (scalding), cooking flames, and sometimes due to exposure to chemicals, electricity, and ionizing radiation [3]. Systematic reviews summarizing studies conducted in the South East Asian subcontinent have identified young age, female gender, poor socioeconomic status and low educational level as major risk factors for burn related injuries and death [4,5,6]. Some studies have also shown that adolescents and younger adults are at even higher risk of suffering from burn injuries in LMIC, compared to children [7]. Unlike HICs, where preventive measures, first aid and burn care management have been instrumental in curbing burn associated disabilities and fatalities, LMICs are still struggling to address the prevention and management of burn injuries in an efficient manner [8]. In many LMICs, individuals still prefer to treat burn injuries at home (using various mixtures of urine and mud, cow dung, beaten eggs, etc.), thus delaying presentation to a health care facility. Limited resources and dearth of trained personnel in health care facilities also pose major challenges in the appropriate treatment of burn injuries in LMIC [3,8].

Bangladesh is no exception to the burn scenario in South East Asia. Almost 173,000 children in Bangladesh suffered from burn injures in 2003, making it the 5th leading cause of childhood illness in the country [9]. Low socioeconomic status, illiteracy or low educational level, crammed housing spaces, and certain cultural practices are shown to increase risk for burn injuries in LMIC settings [10,11]. Additional risk factors are decreased parental supervision for young children; man-made cotton textiles for clothing, and major female involvement in the kitchen, which explains higher, burn morbidity among females than males [12].

Most data available to indicate the burden of death and disability due to burns in Bangladesh are based on small population-based surveys or hospital registries [5,9]. There is a lack of recent large-scale, population-based data that could describe the burden of burn related injuries in Bangladesh and subsequently identify the issues contributing to burn mortality and morbidity. This paper aims to provide an indication of the epidemiology of mortality and morbidity due to burn injuries across all population demographics in rural Bangladesh and re-evaluate the risk factors associated with the risk of burn related disabilities and fatalities using data available from a large population level baseline survey conducted in rural Bangladesh in 2013.

## 2. Materials and Methods

A baseline census was conducted in 2013 as part of an injury prevention intervention study, “Saving of Lives from Drowning (SoLiD)” in seven sub-districts (Upazilas) of rural Bangladesh to establish epidemiological characteristics of fatal and non-fatal injuries [13]. The sub-districts were purposively selected because of their higher risk for childhood drowning. The study was implemented with support from two local organizations, Center for Injury Prevention and Research, Bangladesh (CIRPB) and International Center for Diarrheal Disease Research, Bangladesh (icddr,b). The seven selected sub-districts were Raiganj, Sherpur, Manohardi, Matlab South, Matlab North, Daudkandi, and Chandpur Sadar.

The census covered 993 villages, 270,387 households and a population of approximately 1.2 million in 51 Unions of the seven selected sub-districts. Household members were interviewed on a one-on-one basis (typically one member responded on behalf of one household) to retrieve required information using questionnaires developed and tested for the SoLiD study. Data collection was done in two stages. The first round collected general demographic information on all members of a household as well as any record of injury in the past six months and deaths in the past one year. If an individual reported a particular injury mortality or morbidity event during the first round of data collection, an injury specific form was used to obtain detailed information about the injury and death in a second round of data collection. Injury was defined as any external harm resulting from an assault, fall, cut, burn, animal bite, poisoning, transportation of goods and persons, operating machinery, blunt objects, suffocation, and (near) drowning resulting in the loss of one or more days of normal daily activities, schools, or work [13].

All records of fatal and non-fatal burn injuries were retrieved from the primary database for current analysis. All data were de-identified. Fatal and non-fatal burn injuries were analyzed separately. Descriptive analyses were conducted for demographic and socioeconomic characteristics. For burn injuries, summary statistics were presented based on location (home, work place, and others), intention (unintentional, assault, violence or suicide), source of injury (flame, hot liquid/solid, explosive or chemical) and condition of the individual immediately after an injury. Analyses were also carried out to describe the duration and severity of disability due to burn injury. Both mortality and morbidity rates have been reported.

Unadjusted and adjusted logistic regression models were run to describe the risk factors of non-fatal burn injuries in rural Bangladesh. The variables used in the logistic regression models were gender, age, educational level, occupation, marital status and socio-economic status (SES). Unadjusted logistic regression models used each of these variables as a single predictor for non-fatal burn injuries. Adjusted logistic regression model included all variables in one model. Age was categorized into eight groups. Sex was considered as a binary predictor (male as reference group). Educational, occupation and marital status were categorical variables and SES was considered as five ordinal categories (from lowest to highest).

Age was found to modify the odds of having a burn injury with regards to other variables. Thus additional analyses were conducted for children under nine years of age and individuals 10 years and older separately to adjust for the modification. For children under 10 years of age, adjusted logistic regression model considered only age, gender and socio-economic status. (Supplementary Table S1) Results have been presented for individuals above 10 years of age. Estimated odds ratios for the odds of burn injury have been presented.

### Ethical Statement

All subjects gave informed consent for inclusion before they participated in the study. The study was conducted in accordance with the Declaration of Helsinki, and the protocol was approved by the Ethics Committee of Johns Hopkins Bloomberg School of Public Health, Center for Injury Prevention and Research, Bangladesh and International Center for Diarrheal Disease and Research, Bangladesh. Ethical approval was provided by the Johns Hopkins Bloomberg School of Public Health (approval code—00004746).

## 3. Results

The baseline survey covered a total population of 1,169,594 in the seven sub-districts of rural Bangladesh (Table 1). The overall population comprised of about 51% females (*n* = 601,919). About 43% (508,059) of the study population was 25 to 64 years of age. Approximately 35% (*n* = 409,923) received education at primary level. Almost 35% (*n* = 408,583) of the study population was either retired, unemployed, or was housewives. The majority of study participants were married (49%, *n* = 571,206). The population was almost equally distributed across the SES quintiles (lowest 18%, low 19%, middle 20%, high 21% and highest 22%).

### 3.1. Fatal Burn Mortality Characteristics

There were 25 deaths due to burn injuries among the population surveyed, with a mortality rate of 21 deaths per 1,000,000 people (95% CI: 14–32 per 1,000,000) (Table 1).

**Figure 1:**
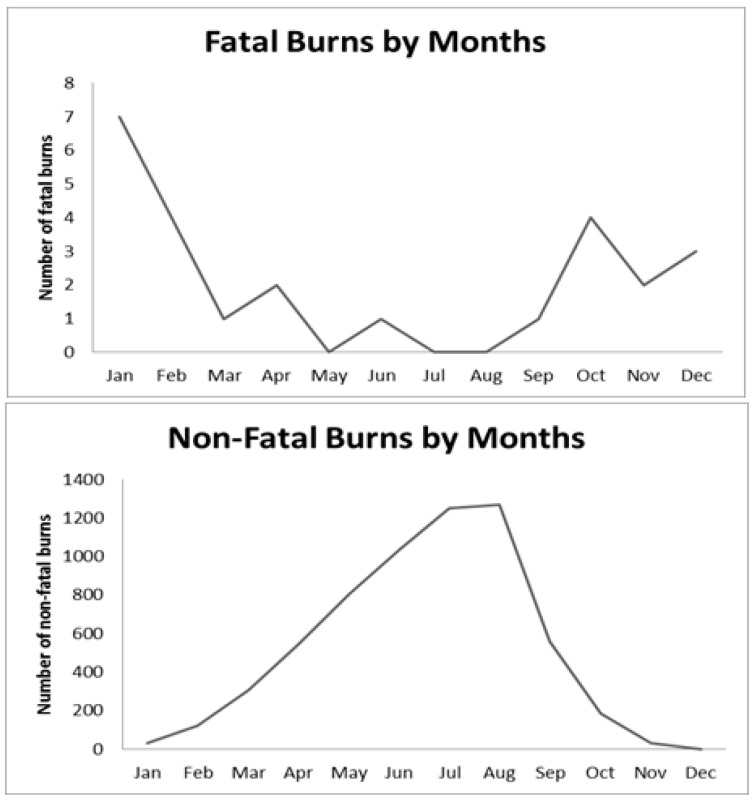
Fatal and non-fatal burn injuries across the months.

Deaths due to burn injuries (92%) were predominantly seen in females, and were significantly higher than deaths in males. Elderly people above 65 years of age bear the burden of burn related deaths. Burns were most commonly seen in individuals who did not have any formal education (68%). Retired or unemployed individuals as well as housewives suffered the highest proportion (88%, *N* = 22) of burn deaths among other employments. Low (36%) and middle-income (24%) households had the highest burden of fatal burn injuries (Table 1). Deaths due to burns were seen mostly in the winter months (Figure 1). Over half (56%) of the injuries occurred in the kitchen. More than half (52%) of the deaths occurred in the home while 36% occurred in the hospital. Flame injuries (88%) were most common, various sources of flame being cooking fire, heating fire and the kerosene lamp. Two deaths occurred as a result of coming in contact with hot cooking and bathing water (Table 2).

### 3.2. Non-Fatal Burn Morbidity Characteristics

**Figure 2:**
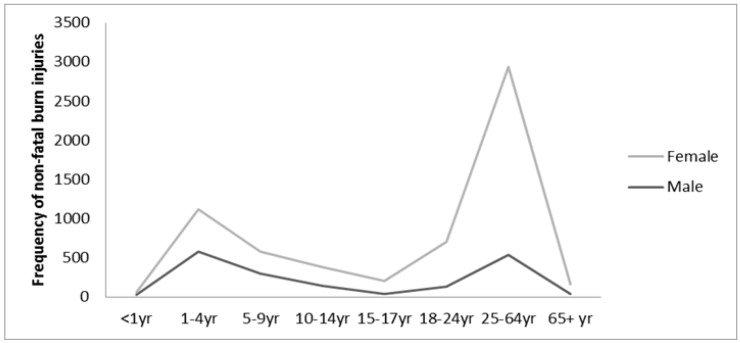
Distribution of Non-fatal burn injuries across age and gender.

A total of 6142 burn related incidents occurred among the approximately 1.2 million people surveyed. The morbidity rate due to burn injuries was 529 injuries per 100,000 populations (95% CI: 517–542 per 100,000). As seen with burn related deaths, more females (71%) had non-fatal burn injuries than males. Higher propensity for burn injuries was seen in children 1 to 4 years of age (18.25%), and adults 25 to 64 years of age (48%), across both sexes (Figure 2). Similar to observations regarding fatal burn injuries, retired or unemployed individuals and housewives had higher prevalence of non-fatal burn injuries, accounting for almost half of all burn related non-fatal outcomes. Almost half of all burn injuries occurred in married people. Non-fatal burn injuries were almost equally distributed across all socioeconomic strata. Unlike deaths due to burns, non-fatal burn injuries occurred mainly in the summer months, with highest propensity in the months of July and August (Figure 1). Almost all burns were unintentional and more than a third (78%) occurred in the kitchen. Contact with hot liquids causing scalds (57%), such as cooking oil appeared to cause the greatest number of burn injuries. Other sources were flame (25%), contact with hot objects (16%), explosives and chemicals. The majority of the cases occurred while preparing food for the household (Table 2).

An injury severity index was calculated, using indicators such as anatomic and physiologic profiles of an injury, post injury immobility, post-injury hospitalization, surgical treatment, post-injury disability, number of days an individual required assistance, and the number of days lost at work or school. Each of these indicators was classified as binary variables. A principle component analysis (a linear combination of a set of variables to explore the underlying structure of those variables as pertaining to a common factor or factors) was applied to summarize the eight indicators into a single index of severity. The scores for all recorded non-fatal injuries were categorized into tertiles—low, medium and high. Results showed that 71% of burn cases had low severity. Only 4% of the individuals had been severely injured by burns [14] (Table 3).

Compared with children 10–14 years of age, adolescents and young adults 15 to 24 years of age had higher odds of sustaining non-fatal burn injuries. Adults 25 to 64 years of age were twice (95% CI: 1.97–2.44) as likely to have non-fatal burn injuries compared with 10–14 years olds. Female participants had more than three times higher chance of burn injuries compared to males (OR = 3.62, 95% CI: 3.36–3.89). The higher the level of education, the lower the risk was for sustaining a non-fatal burn injury. When compared to married people, widowed and never married individuals were at lesser odds of sustaining a burn related injury. Individuals from the lowest socioeconomic level had significantly higher odds to suffer from a burn injury than any other socioeconomic strata (Table 4).

Adjusted logistic regression including all variables (age, sex, education, occupation, marital status and socioeconomic quintiles) showed that the level of education, occupation and socio-economic status was not significantly associated with an amplified risk of burn injury, holding all other factor constant. The ORs did not change meaningfully for age, gender, and marital status while holding other covariates constant. Compared with their respective reference groups, female gender (OR 3.18), and marital status (being married) were found to be at higher odds for non-fatal burn injuries. The lowest SES quintile had higher odds of facing a burn injury when compared with all other four SES quintiles, while adjusting for other covariates (Table 4). Children 1 to 4 years of age were 4.4 (95% CI: 3.37–5.63) times likelier to sustain burn injuries than infants. There was no gender difference in the risk of sustaining a burn injury for children under 9 years of age. Similar association was seen between the risk of sustaining burn injury and socio-economic status among children under 9 years of age as was seen with older individuals, i.e., the higher the socio-economic status, the lower the risk of sustaining a burn injury. Multivariate regression analysis showed increasing age and lower socio-economic status as associated with higher risk of sustaining a burn injury. Gender had no significant association (Supplementary Table S1).

## 4. Discussion

Based on this large-scale cross-sectional data, the mortality rate due to burn injuries was 21 per 1,000,000 populations in rural Bangladesh. The majority of fatalities were seen in children between one and four years of age and the elderly above 65 years of age, as has been reported in previous studies from South Asia [15,16]. Similarly, burn injury mortality was higher among female participants in our study [17,18,19]. Non-fatal burn injury is quite high in this population (528 people in every 100,000 population).

The study highlighted the disproportionate distribution of non-fatal injuries in select Upazilas in Bangladesh. This finding came as a surprise for the research team as well and on further exploration, no substantial evidence could be found to support the imbalance. This leaves scope for future research work to study these variations, which could possibly generate some best practices from Upazilas to be translated into others with a higher proportion of burn injuries.

Most fatal burn injuries occurred in the winter season. The seasonal variation seen in the distribution of fatal and non-fatal burn injuries can be related to the need for prolonged heating during the winter months. When used for long durations of the night, heat sources such as coal and wood put inhabitants at higher risk of death due to burns [20,21,22]. Non-fatal injuries displayed different seasonal patterns, and injuries were high during the summer and monsoon months. This is likely attributed to domestic, or occupational activities, such as cooking or working with machinery, that have shorter contact spans and a lower threshold to cause burn deaths. When it comes to location, most burn incidents occurred in the home, mainly in the kitchen. Flames and scalds were the most common cause of burns deaths and non-fatal injuries respectively. Similar results were seen in previous studies from Bangladesh and surrounding countries such as India, Sri Lanka, Pakistan, and Nepal possibly due to the use of unsafe cooking stoves with open fire and lack of safe practice of fuels such as petroleum and butane across regions of South Asia [16,23,24,25,26,27,28]. A study conducted in Bangladesh covering both urban and rural populations reported electrical burns to be the most common cause of burn injuries [29]. However, since this study was strictly limited to rural regions and did not explore electrical burns, there is scope for further research to tease out the generalized burden of burn injuries in Bangladesh.

Female participants were twice as likely to suffer from burn injuries across all age groups. A study showed that even though boys were more vulnerable to unintentional injuries across all ages, girls were more susceptible to fire related injuries [30]. Similar results as seen in this study were observed in various parts of India, Pakistan, Sri Lanka and Nepal [15,28,31,32]. Cultural and societal norms denote females as majorly responsible for preparing meals in kitchens, which makes them more susceptible to fire injuries. Also, loose clothing, floor level cooking and crammed housing spaces increase risk for sustaining burn injuries among women [11,33].

The lowest socio-economic level was seen to sustain most burn injuries. Another study from Bangladesh showed that children from poor families were almost 3 times more likely to die following a burn injury as compared to children from other socio-economic levels [30]. A systematic review studying various socio-economic factors for the risk of burn injuries reported low income, lack of education, unemployment, crowded and sub-standard living situations, among others to increase risk of burn injuries [34]. In addition, injury severity due to burns has been reported to be higher with reduced SES [35].

This study provides valuable information in terms of analyzing and describing burn injuries in rural Bangladesh. To our knowledge, most studies conducted in LMICs are based on hospital registries and police records. It is likely that a large number of burn mortality and morbidity are not recorded. By conducting a large-scale, household-level survey, this current study is much more representative of the true burden, thus less likely to underestimate the burden of burn injury. Household-level surveys also helped account for bias incurred by facility-based surveys, such as reporting bias, bias in age groups, and bias induced by SES.

The study also has some limitations. This set of analysis is based on cross-sectional data, therefore cannot support causal association. The covariates selected for regression models were based on program implementation experience and previous literature. This study still cannot pinpoint ‘true’ causes of burn injuries, but rather, helps identify potential determinants. Second, despite including seven Upazilas, the results still need to be interpreted with caution when it comes to external validity.

However, since the study covered predominantly rural locations in Bangladesh, the results may not be applicable to the urban Bangladeshi population. Studies need to be conducted in both urban and rural settings to measure variations in the burden of mortality and morbidity due to burn injuries.

## 5. Conclusions

Rural Bangladesh faces a major public health concern in the face of significant prevalence of burn related mortalities and morbidities. Young children and adults are more at risk of sustaining a burn related injury. Females across all age groups are more prone to have a burn injury in their lives. The kitchen environment at homes is most commonly the place of a burn incident. Modifications in a kitchen setup, use of safe cook-stoves and heating sources, complete barricading of the cooking area to prevent contact with children and burn injury prevention educational programs for women are some relevant interventions that may be implemented in rural Bangladesh with involvement from communities to reduce burn related accidents and deaths and thereby reduce the loss of subsequent school and work hours. Age-targeted interventions, awareness of first aid protocols, and improvement of acute care management would also be potential leads to curb death and disability due to burn injuries.

## Figures and Tables

**Table 1:** Socio-demographic characteristics of fatal and non-fatal burns injury patients.

Characteristics	All Population (*N* = 1,169,594)		Fatal Burn Injuries (*N* = 25)		Non-Fatal Burns (*N* = 6142)	
	*N*	(%)	*N* (%)	Mortality Rate/1,000,000	*N* (%)	Morbidity Rate/100,000
*Upazila*						
Matlab North	265,897	(22.7)	5 (20.0)	18.8	1019 (16.6)	385.3
Matlab South	209,772	(17.9)	3 (12.0)	14.3	1103 (18.0)	528.6
Chadpur Sadar	128,356	(11.0)	0 (0.0)	0	347 (5.7)	271.6
Raiganj	104,357	(8.9)	6 (24.0)	57.5	932 (15.2)	898.2
Sherpur	228,519	(19.5)	6 (24.0)	26.3	1378 (22.4)	606.1
Manohardi	204,319	(17.5)	4 (16.0)	19.6	1150 (18.7)	566.2
Daud Kandi	28,373	(2.4)	1 (4.0)	35.2	213 (3.5)	755.8
*Sex*						
Male	567,674	(48.5)	2 (8.0)	3.5	1787 (29.1)	316.5
Female	601,919	(51.5)	23 (92.0)	38.2	4355 (70.9)	727.5
*Age*						
<1 year	22,141	(1.9)	0 (0.0)	0	92 (1.0)	427.4
1–4 years	90,523	(7.7)	0 (0.0)	0	1121 (18.3)	1243.3
5–9 years	139,728	(12.0)	0 (0.0)	0	574 (9.4)	411.1
10–14 years	142,121	(12.2)	1 (4.0)	7.0	377 (6.1)	265.4
15–17 years	62,098	(5.3)	1 (4.0)	16.1	208 (3.4)	335.2
18–24 years	133,534	(11.4)	4 (16.0)	29.9	701 (11.4)	525.5
25–64 years	508,059	(43.4)	6 (24.0)	11.8	2932 (47.7)	579.2
65+ years	71,389	(6.1)	13 (52.0)	182.1	167 (2.7)	244.6
*Education*						
No education	295,314	(25.3)	17 (68.0)	230.3	1538 (25.0)	526.8
Primary	407,923	(34.9)	4 (16.0)	39.2	1753 (28.5)	430.8
Secondary	289,658	(24.8)	3 (12.0)	41.4	1443 (23.5)	499.3
A levels	45,618	(3.9)	0 (0.0)	0	176 (2.9)	386.7
College	13,526	(1.2)	0 (0.0)	0	38 (0.6)	281.8
Advanced/professional degree	4729	(0.4)	1 (4.0)	845.8	11 (0.2)	234.0
Not applicable (Under 5)	112,664	(9.6)	0 (0.0)	0	1183 (19.3)	1059.2
*Occupation*						
Agriculture	104,956	(9.0)	1 (4.0)	38.1	234 (3.8)	225.4
Business	61,661	(5.3)	0 (0.0)	0	147 (2.4)	239.7
Skilled labor (Professional)	89,151	(7.6)	1 (4.0)	44.9	263 (4.3)	296.0
Unskilled/domestic (Unskilled)	24,520	(2.1)	0 (0.0)	0	72 (1.2)	295.1
Rickshaw/bus (Transport worker)	17,037	(1.5)	0 (0.0)	0	47 (0.8)	276.6
Students	312,537	(26.7)	1 (4.0)	12.8	922 (15.0)	295.2
Retired/unemployed/housewife	408,583	(35.0)	22 (88.0)	215.4	3059 (49.8)	754.1
Not applicable (children)	144,454	(12.4)	0 (0.0)	0	1369 (22.3)	954.6
Not applicable (others)	5948	(0.5)	0 (0.0)	0	28 (0.5)	484.3
*Marital status*						
Married	571,206	(48.8)	11 (44.0)	77.0	3358 (54.7)	591.5
Never married	227,319	(19.4)	3 (12.0)	52.8	622 (10.1)	273.9
Divorced	3220	(0.3)	0 (0.0)	0	8 (0.1)	250.2
Widowed	53,096	(4.5)	10 (40.0)	753.4	239 (3.9)	462.8
Separated	2717	(0.2)	0 (0.0)	0	16 (0.3)	592.2
Others (children under 12)	312,035	(26.7)	1 (4.0)	12.8	1899 (30.9)	610.7
*SES quintiles*						
Lowest	211,601	(18.1)	4 (16.0)	18.9	1254 (20.4)	596.4
Low	218,695	(18.7)	9 (36.0)	41.1	1166 (19.0)	535.8
Middle	238,371	(20.4)	6 (24.0)	25.2	1199 (19.5)	505.6
High	247,716	(21.2)	3 (12.0)	12.1	1258 (20.5)	510.5
Highest	253,210	(21.7)	3 (12.0)	11.9	1265 (20.6)	502.3

**Table 2:** Distribution of fatal and non-fatal burns injuries.

Characteristics	Fatal Injuries		Non-Fatal Injuries	
	*N*	(%)	*N*	(%)
*Place of injury*				
Bedroom	7	28	273	4.44
Kitchen	14	56	4385	71.39
Veranda	1	4	32	0.52
Yard	3	12	729	11.87
Others	-	-	644	11.78
*Item causing injury*				
Flame	22	88	1555	25.32
Hot liquid	2	8	3472	56.53
Hot object	0	0	1003	16.33
Explosive	0	0	19	0.31
Chemical	0	0	30	0.49
Other	1	4	63	1.03
*Place of death*				
Home	13	52	-	-
On the way to hospital ^1^	1	4		
Hospital	9	36		
On the road ^2^	2	8		

^1^ died while being transported to a hospital; ^2^ did not receive any form of emergency medical assistance.

**Table 3:** Injury severity index for non-fatal burn injuries.

Injury Severity	Non-Fatal Injuries	
	*N*	(%)
Low	4283	69.73
Medium	1499	24.41
High	245	3.99

**Table 4:** Unadjusted and adjusted analysis of non-fatal burn injury by age group, sex, education, occupation, marital status and SES quintiles.

Characteristics	Unadjusted			Adjusted		
	OR	95% CI	*p* Value	OR	95% CI	*p* Value
*Age group*						
10–14 years	Reference group			Reference group		
15–17 years	1.26	1.07–1.49	<0.001	1.08	0.89–1.32	0.381
18–24 years	1.98	1.75–2.25	<0.001	1.05	0.86–1.29	0.618
25–64 years	2.18	1.97–2.44	<0.001	1.02	0.83–1.27	0.830
65+ years	0.92	0.77–1.11	0.378	0.52	0.39–0.68	<0.001
*Sex*						
Male						
Female	3.62	3.36–3.89	<0.001	3.18	2.83–3.58	<0.001
*Education*						
No education						
Primary	0.79	0.73–0.85	<0.001	0.87	0.80–0.94	0.001
Secondary	0.91	0.84–0.98	0.012	0.92	0.84–0.99	0.049
A levels	0.71	0.60–0.82	<0.001	0.86	0.73–1.03	0.105
College	0.51	0.37–0.71	<0.001	0.88	0.49–0.95	0.025
Advanced/professional degree	0.43	0.24–0.76	0.005	0.60	0.33–1.10	0.102
*Occupation*						
Agriculture						
Business	1.06	0.86–1.29	0.51	1.06	0.87–1.32	0.562
Skilled labor (Professional)	1.31	1.10–1.56	0.003	1.19	0.99–1.43	0.062
Unskilled/domestic (Unskilled)	1.29	0.99–1.69	0.06	1.11	0.85–1.46	0.420
Rickshaw/bus (Transport worker)	1.22	0.89–1.67	0.21	1.19	0.87–1.63	0.269
Students	3.37	2.95–3.85	<0.001	1.12	0.72–1.14	0.407
Retired/unemployed/housewife	1.42	20.79–2.54	0.25	0.91	1.01–1.43	0.035
*Marital status*						
Married						
Never married	0.46	0.42–0.50	<0.001	0.65	0.55–0.76	<0.001
Divorced	0.42	0.21–0.84	0.02	0.34	0.17–0.67	0.002
Widowed	0.78	0.69–0.89	<0.001	0.68	0.58–0.78	<0.001
Separated	1.00	0.61–1.64	1.00	0.78	0.47–1.28	0.334
Others (children 10–12)	0.41	0.34–0.48	<0.001	0.58	0.45–0.76	<0.001
*SES quintiles*						
Lowest						
Low	0.94	0.85–1.04	0.01	0.98	0.89–1.08	0.685
Middle	0.93	0.84–1.02	<0.001	0.96	0.87–1.06	0442
High	0.94	0.79–0.92	<0.001	0.98	0.89–1.04	0.745
Highest	0.95	0.78–0.91	<0.001	0.98	0.89–1.08	0.687

## Supplementary Materials

The following are available online at www.mdpi.com/1660-4601/14/4/381/s1, Table S1: Unadjusted and adjusted analysis of non-fatal burn injury by age group, sex and SES quintiles for children under 9 years of age.

## Abbreviations

LMIC	low and middle income countries
HIC	high income countries
CIPRB	Center for Injury Prevention and Research, Bangladesh
Icddr,b	International Center for Diarrheal Disease Research, Bangladesh
CI	Confidence Interval
OR	Odds ratio
