# Supplementary material for: Epidemiology of Burns in Rural Bangladesh: An Update
Source: Int J Environ Res Public Health. 2017 Apr 5;14(4):381. doi: 10.3390/ijerph14040381 (PMC5409582; doi:10.3390/ijerph14040381)
Supplement: Supplementary file 1 [file ijerph-14-00381-s001.pdf]

Int. J. Environ. Res. Public Health **2017**, *14*, x; doi: www.mdpi.com/journal/ijerph

Supplementary Materials: Epidemiology of Burns in Rural Bangladesh: An Update

Siran He, Olakunle Alonge *, Priyanka Agrawal, Shumona Sharmin, Irteja Islam, Saidur Rahman Mashreky and Shams El Arifeen

Table S1: Unadjusted and adjusted analysis of non-fatal burn injury by age group, sex and SES quintiles for children under 9 years of age.

	**Unadjusted**			**Adjusted**		
	**OR**	**95% CI**	**P value**	**OR**	**95% CI**	**P value**
Age group						
<1 yr	Reference group			Reference group		
1–4 yrs	4.36	3.37–5.63	<0.001	4.31	3.34–5.58	<0.001
5–9 yrs	1.41	1.09–1.85	<0.001	1.41	1.08–1.83	0.011
Sex						
Male	Reference group			Reference group		
Female	1.00	0.91–1.1	0.981	1.00	0.91 – 1.09	0.980
SES quintiles						
Lowest	Reference group			Reference group		
Low	0.85	0.74–0.97	0.018	0.86	0.75–0.99	0.041
Middle	0.73	0.63–0.84	<0.001	0.74	0.64–0.85	<0.001
High	0.74	0.64–0.84	<0.001	0.74	0.64–0.85	<0.001
Highest	0.67	0.58–0.78	<0.001	0.66	0.57–0.76	<0.001

© 2017 by the authors. Submitted for possible open access publication under the terms and conditions of the Creative Commons Attribution (CC BY) license (http://creativecommons.org/licenses/by/4.0/).
